# Hyaluronidase Enhances Targeting of Hydrogel-Encapsulated Anti-CTLA-4 to Tumor Draining Lymph Nodes and Improves Anti-Tumor Efficacy

**DOI:** 10.3390/gels8050284

**Published:** 2022-05-03

**Authors:** Airi Harui, Michael D. Roth

**Affiliations:** Division of Pulmonary & Critical Care Medicine, Geffen School of Medicine at UCLA, Los Angeles, CA 90095-1690, USA; mroth@mednet.ucla.edu

**Keywords:** checkpoint inhibitor blockade, immunotherapy, hydrogel, anti-CTLA-4, peri-tumor injection, hyaluronidase, antibody delivery, tumor draining lymph nodes

## Abstract

Immunotherapy targeting checkpoint inhibitors, such as CTLA-4 and/or PD-1, has emerged as a leading cancer therapy. While their combination produces superior efficacy compared to monotherapy, it also magnifies inflammatory and autoimmune toxicity that limits clinical utility. We previously reported that a peri-tumor injection of low-dose hydrogel-encapsulated anti-CTLA-4 produced anti-tumor responses that were equal to, or better than, systemic dosing despite a >80% reduction in total dose. Injection of hydrogel-encapsulated anti-CTLA-4 was associated with low serum exposure and limited autoimmune toxicity, but still synergized with anti-PD-1. In this report, we employ live and ex vivo imaging to examine whether peri-tumor administration specifically targets anti-CTLA-4 to tumor-draining lymph nodes (TDLN) and whether the incorporation of hyaluronidase enhances this effect. Tumor-free survival analysis was also used to measure the impact of hyaluronidase on tumor response. Compared to systemic dosing, peri-tumor injection of hydrogel-encapsulated anti-CTLA-4/DyLight 800 resulted in preferential labeling of TDLN. Incorporating hyaluronidase within the hydrogel improved the rapidity, intensity, and duration of TDLN labeling and significantly improved tumor-free survival. We conclude that hydrogel-encapsulated anti-CTLA acts as a localized antibody reservoir and that inclusion of hyaluronidase optimizes the blockade of CTLA-4 in TDLN and thereby imparts superior anti-tumor immunity.

## 1. Introduction

Monoclonal antibodies (mAbs) that block immune checkpoint inhibitors are rapidly expanding the treatment options for patients with soft tissue cancers [1,2]. However, individual mAbs are effective in only a minority of patients while combination therapy that blocks both CTLA-4 and PD-1 yields a significantly greater response and progression-free survival [3,4,5,6,7]. Unfortunately, systemic administration of combination therapy also increases acute inflammatory and autoimmune toxicity that can limit clinical utility [8,9]. Hydrogel-encapsulated checkpoint inhibitor therapy has recently been identified as an approach for improving this narrow benefit-to-risk ratio [10,11,12,13]. Using a mouse model, we previously reported that peri-tumor injection of a controlled-release hydrogel containing low-dose anti-CTLA-4 produces equal or greater tumor control than does high-dose systemic therapy [13]. This targeted low-dose approach still synergizes with systemic anti-PD-1 therapy and promotes lasting systemic protection against tumor re-challenge [13]. Local injection of anti-CTLA-4 alone (without a controlled-release hydrogel) produces only a limited response. Furthermore, using a NOD.H-2h4 model to assess anti-CTLA-4-associated autoimmune thyroiditis, systemic administration of anti-CTLA-4 induced high titers of anti-thyroglobulin antibodies while hydrogel-based administration did not. At the conclusion of that work, we hypothesized that controlled regional delivery of low-dose anti-CTLA-4 results in a sustained perfusion of tumor draining lymph nodes (TDLN) that selectively expands and activates tumor-specific T cells while sparing autoimmune T cell activation at distant nodal sites. In related work, we demonstrated that the addition of hyaluronidase (HAse) to the hydrogel allowed us to fine tune the rate of mAb release and, by promoting autolysis of the hydrogel reservoir, facilitates repeated administration at the same location [14]. 

In the current brief report, we evaluate whether the incorporation of HAse into the hydrogel matrix has other beneficial effects on therapy. Injection of recombinant human HAse is approved by the FDA to permeabilize soft-tissue matrix in order to promote the uptake of fluids, drugs, and mAb therapies through existing lymphatic pathways [15]. Given our focus on regional perfusion of TDLN with anti-CTLA-4, we hypothesized that HAse might enhance lymphatic uptake and nodal targeting of administered anti-CTLA-4 and therefore promote even greater anti-tumor efficacy. In this study, live animal imaging is used to track anti-CTLA-4 distribution following either systemic injection, targeted peri-tumor injection of a standard hydrogel, or administration of a hydrogel that contains incorporated HAse. Lymph nodes were also excised and imaged ex vivo. Our established pre-clinical tumor immunotherapy model was then employed to evaluate the effects of HAse on anti-tumor activity and tumor-free survival [13]. 

## 2. Results and Discussion

### 2.1. Delivery of Hydrogel-Encapsulated Anti-CTLA-4 by Peri-Tumor Injection Preferentially Targets TDLN

**Figure 1:**
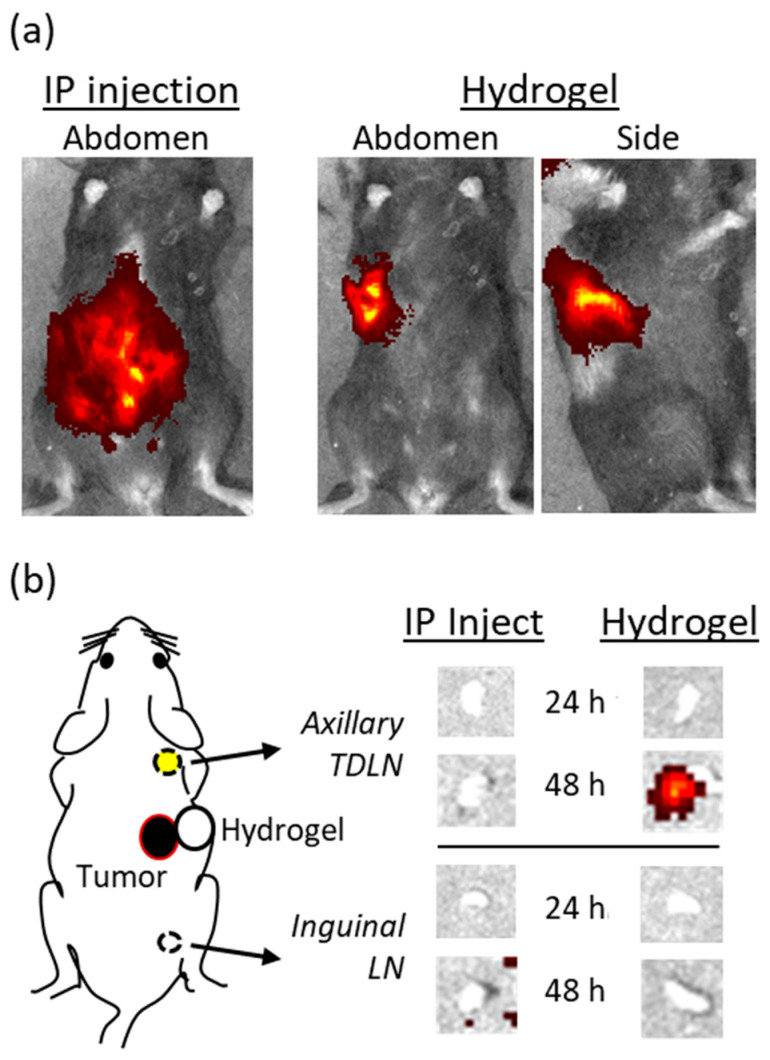
Biodistribution of fluorescent-labeled anti-CTLA-4 to tumor draining lymph nodes (TDLN) following either systemic (intraperitoneal; IP) or hydrogel (subcutaneous; SQ) injection. C57BL/6 mice bearing palpable MC-38 tumors implanted in the right posterior flank were treated with either 100 µg of anti-CTLA-4/DyLight 800 by IP injection or 25 µg of hydrogel-encapsulated anti-CTLA-4/DyLight 800 by peri-tumor SQ injection. Biodistribution of the injected anti-CTLA-4 was determined (**a**) immediately following administration by whole-animal in vivo optical fluorescence imaging and (**b**) at 24 and 48 h by ex vivo optical fluorescence imaging performed on surgically resected axillary TDLN and inguinal LN.

A whole-animal optical imaging technique was adapted to investigate whether a peri-tumor injection of hydrogel-encapsulated anti-CTLA-4 creates a subcutaneous (SQ) mAb reservoir that preferentially targets regional TDLN. A near-infrared anti-CTLA-4/DyLight 800 conjugate was developed in order to allow both in vivo live imaging and ex vivo quantitation of mAb trafficking. When tumor-bearing mice were treated with 100 µg of anti-CTLA-4/DyLight 800 by intraperitoneal (IP) injection, the labeled mAb rapidly dispersed throughout the peritoneal cavity as expected (Figure 1a). In contrast, a peri-tumor SQ injection of low-dose hydrogel-encapsulated anti-CTLA-4/DyLight 800 (25 µg) resulted in a localized reservoir of mAb at the site of injection. These primary distribution patterns remained relatively constant when serially imaged over the course of 48 h. Expression of the label within lymph nodes was not observed in live animals, likely due to the overwhelming intensity of fluorescence from the primary injection and the deeper location of lymph nodes. In order to improve the sensitivity and specificity for detecting the micro-distribution of anti-CTLA-4 DyLight 800, sets of animals that had been treated in an identical manner were sacrificed at 24 and 48 h after injection. Bilateral axillary and inguinal lymph nodes (LN) were surgically excised and simultaneously examined by ex vivo optical imaging (Figure 1b). No fluorescent signal was detected at 24 h in any LN regardless of whether the animals received IP or SQ (hydrogel-based) dosing with anti-CTLA-4/DyLight 800. However, at 48 h, a fluorescent signal was always observed in axillary TDLN from animals that had received low-dose hydrogel-encapsulated anti-CTLA-4/DyLight 800. No signal was detected in animals that had received IP dosing even though they had received four-times the mAb dose. In addition, no fluorescent signals were detected from inguinal or contralateral LN in either group. These findings directly support our primary hypothesis that encapsulating anti-CTLA-4 within the hydrogel matrix and delivering it by peri-tumor injection produces a localized reservoir of mAb that preferentially targets TDLN. Minimizing systemic exposure to anti-CTLA-4 is particularly important due to the role of CTLA-4 in autoimmune-related toxicity [16]. As demonstrated previously, high-dose systemic administration of anti-CTLA-4 results in high serum concentrations that trigger/enhance the production of autoimmune antibodies [13]. These results also explain why targeted low-dose anti-CTLA-4, as delivered by the hydrogel, can produce equal/greater anti-tumor efficacy than does systemic dosing [13].

### 2.2. Incorporation of HAse into the Hydrogel Matrix Enhances Antibody Delivery to TDLN

We had previously reported that incorporating HAse into the hydrogel mixture results in a number of beneficial effects. The reactive moieties on thiolated carboxymethyl hyaluronic acid (CMHA-S) and poly-(ethylene glycol)-diacrylate (PEG-DA), designed to promote spontaneous cross-linking and formation of the hydrogel matrix, can also interact with incorporated proteins with denaturing effects. The presence of HAse reduces this effect, likely by acting as an alternative protein target or impairing the interaction of these reactive moieties with incorporated proteins [11]. This protective effect from HAse was also observed for incorporated anti-CTLA-4. In an in vitro antibody release assay, total recovery of anti-CTLA-4 from the standard hydrogel preparation was 86.8% and this increased to 93.6% with addition of HAse 50 U and 97.8% with HAse 250 U (Figure S1). In addition, incorporated HAse breaks down the hyaluronic acid backbone of the matrix in a dose- and time-dependent manner. This can be used to fine-tune the rate of mAb release from the hydrogel matrix and assures complete release of encapsulated mAb payloads [14,17] (Figure S1). HAse also promotes more rapid resorption of the hydrogel matrix, promoting repeated injections at the same site for future cycles of immunotherapy. 

**Figure 2:**
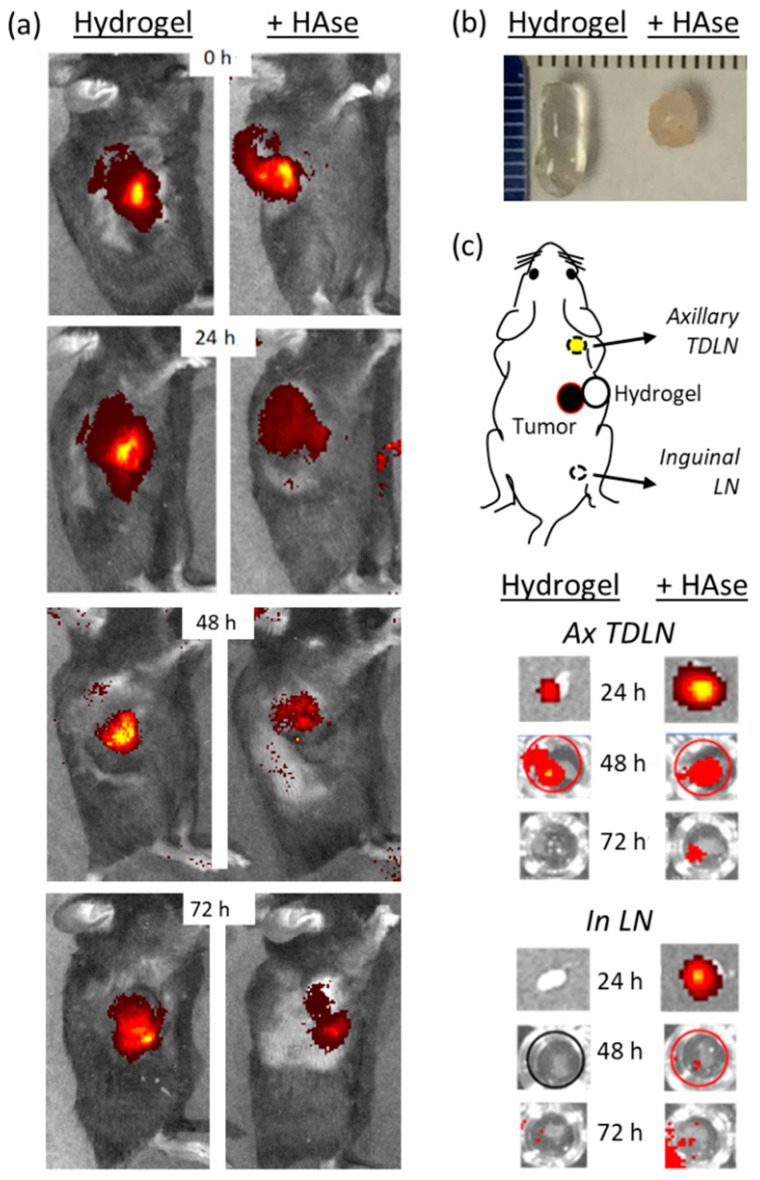
Impact of hyaluronidase (HAse) on the delivery of hydrogel-encapsulated anti-CTLA-4 to TDLN. C57BL/6 mice bearing palpable MC-38 tumors were treated with 50 µg of hydrogel-encapsulated anti-CTLA-4/DyLight 800 by peri-tumor SQ injection. Anti-CTLA-4 biodistribution was compared in animals receiving a standard hydrogel formulation to one that incorporated HAse (250 U). (**a**) Whole-animal in vivo optical fluorescence imaging of the injection site immediately following injection (0 h) and at 24, 48, and 72 h. (**b**) Hydrogels from the same animals were surgically recovered after 2 weeks for visual inspection. (**c**) Axillary TDLN and ipsilateral inguinal LN were surgically resected at 24, 48, and 72 h from a cohort of animals treated in the same manner and subjected to ex vivo optical fluorescence imaging.

HAse is clinically approved for patient use based on its ability to break down SQ tissue barriers and facilitate the access of fluids, medications, and mAb into tissue lymphatic pathways [15,18]. Existing data suggest that this promotes faster and more efficient trafficking to draining LN [19]. We therefore hypothesized that addition of HAse to the hydrogel formulation could enhance targeting and exposure of TDLN to anti-CTLA-4. To test this hypothesis, tumor-bearing mice were injected with hydrogel-encapsulated anti-CTLA-4/DyLight 800 (50 µg) in the presence or absence of 250 U HAse and then imaged for trafficking of the fluorescent label (Figure 2). In vivo live imaging shows similar localization and concentration of the fluorescent label in both groups at the time of injection. However, from 24 through 72 h, there is a significant and time-dependent reduction in the anti-CTLA-4/DyLight 800 signal at the injection site when HAse was incorporated into the injected hydrogels. Our conclusion is that anti-CTLA-4/DyLight 800 is released faster and/or more effectively in vivo when HAse is present. Consistent with this, when implanted hydrogels were recovered by surgical excision at 2 weeks after injection (Figure 2b), the hydrogels containing HAse were dramatically smaller and appeared to be infiltrated by host cells, consistent with accelerated degradation.

The fundamental question addressed in the next study was whether this results in more effective perfusion of TDLN by anti-CTLA-4. As previously described, sets of tumor-bearing animals that had been treated in an identical manner were sacrificed at 24, 48, and 72 h after injection of anti-CTLA-4/DyLight 800. Recovered axillary and inguinal LN were simultaneously examined for the presence of labeled anti-CTLA-4 by ex vivo optical imaging (Figure 2c). As hypothesized, a higher level of fluorescence was detected in axillary TDLN at 24 h when the hydrogel contained HAse. At 48 h, the fluorescence level was similar in both groups while the fluorescent signal was only detected at 72 h from animals that received hydrogel containing HAse. This visual trend was confirmed by measuring total fluorescent emission from LN recovered at these time points (Table S1). Ex vivo imaging of recovered inguinal LN showed a similar—but less intense—pattern in animals that received hydrogels containing HAse but no uptake by inguinal LN at any time point in the absence of HAse (Figure 2c). This interesting finding suggests that the increased tissue permeability resulting from HAse can spread anti-CTLA-4 to a wider tissue distribution. Given the size difference between mice and humans, it is not yet clear whether this would have a meaningful effect in a clinical setting. We did not detect anti-CTLA-4 uptake by other organs by whole-body live imaging, nor was a signal detected in isolated spleens. This likely reflects the imaging threshold of our approach and it is very possible that more sensitive approaches—such as immunohistochemistry or flow cytometry—might yield findings that are beyond the focus of our targeted studies.

Taken together, these results suggest that incorporating HAse into the hydrogel formulation enhances the release of functional anti-CTLA-4 from the hydrogel matrix; opens lymphatic barriers in surrounding SQ tissues; and produces faster, higher, and more prolonged binding to target sites in TDLN while still sparing mAb accumulation in distant (e.g., contralateral) LN. 

### 2.3. Incorporation of HAse into the Hydrogel Matrix Enhances the Anti-Tumor Efficacy of Low Dose Anti-CTLA-4

**Figure 3:**
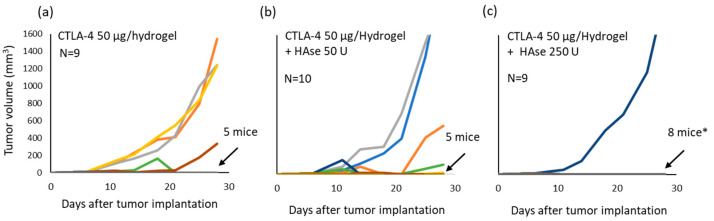
Incorporation of HAse enhances the anti-tumor activity of hydrogel-encapsulated anti-CTLA-4. C57BL/6 mice injected with MC-38 tumor cells were treated with 50 µg of hydrogel-encapsulated anti-CTLA-4 by peri-tumor SQ injection at days 6 and 11 after tumor implantation. Groups of animals received either a standard hydrogel formulation (**a**), or a hydrogel formulated with either 50 U (**b**) or 250 U (**c**) of HAse. Tumor volume was measured every 3 or 4 days over a course of 28 days. Each line represents tumor growth in a single mouse. The number of tumor-free animals at day 28 are indicated (* *p* < 0.05 compared to no HAse control by log-rank test).

Targeting of TDLN occurs when a peri-tumor injection of hydrogel-incorporated anti-CTLA-4 is delivered, and this promotes effective tumor killing while limiting systemic exposure [13]. The addition of HAse improves anti-CTLA-4 release from the hydrogel, promotes mAb access to the lymphatic pathway, and results in higher and more prolonged targeting of CTLA-4 binding sites in TDLN. Anti-CTLA-4 results in an expansion of tumor-reactive T cells, reduction in CTLA-4-expressing cells, and a relative expansion of cytotoxic CD8 cells [13]. The remaining question is whether greater TDLN perfusion by anti-CTLA-4 translates into better therapeutic outcomes. To test the hypothesis that HAse can improve immune activation resulting from low-dose anti-CTLA-4, tumor-bearing mice (N = 9 or 10) were treated with hydrogel-encapsulated anti-CTLA-4 (50 µg) that contained either 0, 50, or 250 U of HAse. Peri-tumor injections were delivered on days 6 and 11 after tumor implantation [13]. Tumor growth was monitored for 28 days (Figure 3). All tumors, regardless of assigned treatment group, grew in the first few days after starting therapy but thereafter the response was significantly different in the treatment group receiving 250 U HAse. At the end of 28 days, 5 mice (56%) in the control group (no HAse) were tumor free; 5 mice (50%) in the group receiving gels containing 50 U HAse were tumor free; but tumor-free survival was present in 8 mice (89%) that had received hydrogels containing 250 U HAse (*p* = 0.04; log-rank test for tumor-free survival; Figure S2). Additional controls demonstrated that these responses required the presence of anti-CTLA-4 as the difference in treatment response was highly significant when comparing animals treated with hydrogels containing only 250 U HAse and those receiving anti-CTLA-4 plus 250 U HAse (*p* < 0.001 by ANOVA; data not shown). The synergism that occurs between the delivery of anti-CTLA-4 and HAse, when delivered together in a peri-tumor injection of self-polymerizing hydrogel, therefore spans from enhanced effects on anti-CTLA-4 release, lymphatic uptake, and targeting of TDLN to a positive impact on anti-tumor efficacy. 

## 3. Conclusions

In this study, a combination of whole-animal in vivo imaging and ex vivo imaging of recovered LNs provided direct evidence that a peri-tumor injection of hydrogel-encapsulated anti-CTLA-4 creates a local drug reservoir that targets mAb delivery to TDLN. While systemic anti-CTLA-4 therapy has the potential to disrupt tumor-associated immunosuppression through multiple pathways and at multiple sites, targeted delivery to TDLN has been shown in animal models to promote proportionally greater T-cell activation and systemic tumor control [11]. These outcomes occur even in the absence of direct perfusion of the tumor or depletion of intratumoral regulatory T cells [11]. This mechanism of action likely explains the capacity for hydrogel-delivered anti-CTLA-4 to induce effective anti-tumor immunity while sparing systemic exposure and drug-associated autoimmune toxicity [13]. Furthermore, we demonstrated that incorporating HAse into the hydrogel facilitates anti-CTLA-4 release, as well as the speed, intensity, and duration of its binding within TDLN. The end result is a significant improvement in systemic anti-tumor efficacy and tumor-free survival without an increase in delivered dose. According to convention, the dose of systemic anti-CTLA-4 therapy is directly linked to both its efficacy and toxicity [20,21]. The capacity to target TDLNs via a peri-tumor injection disrupts this relationship. As demonstrated here, incorporation of HAse into the hydrogel matrix further enhances the effectiveness of low-dose anti-CTLA-4 therapy and should further improve the benefit-to-risk ratio associated with its use. As such, these pre-clinical investigations define a clear pathway for employing hydrogel-encapsulated anti-CTLA-4 as a strategy for improving the tolerability and potency of cancer immunotherapy. As previously noted, the greatest promise from such an approach would be the ability to safely administer combination therapy that included hydrogel-encapsulated anti-CTLA-4, targeting TDLN, and a systemic PD-1 checkpoint inhibitor.

## 4. Materials and Methods

### 4.1. Animals

C57BL/6 mice were purchased from the Charles River Laboratory (Wilmington, MA, USA) and housed at the UCLA Division of Laboratory Animal Medicine facility. All protocols and procedures were approved by the UCLA Animal Research Committee.

### 4.2. Reagents

Mouse colorectal cancer cell line, MC-38, was obtained from the Division of Cancer Treatment and Diagnosis Tumor Repository, National Cancer Institute. Hydrogel matrix components, CMHA-S and PEG-DA, were provided by Lineage Cell Therapeutics (Alameda, CA, USA). The anti-mouse CTLA-4 (clone # 9H10) was purchased from BioXcell (West Lebanon, NH, USA) and a matching fluorescent-conjugated anti-mouse CTLA-4/Dylight800 was prepared specifically for this study by Leinco Technology (St. Louis, MO). Purified bovine hyaluronidase (HAse) was from MP Biomedicals (Santa Ana, CA, USA).

### 4.3. Hydrogel Formulation

Hydrogels were formulated by first mixing the anti-CTLA-4 9H10 clone (50 µg/gel) or the anti-CTLA-4/Dylight 800 fluorescent construct (25 or 50 µg/gel) with the solubilized PEG-DA and then combining with CMHA-S to initiate spontaneous cross-linking as described previously [13]. Briefly, CMHA-S and PEGDA were individually dissolved in degassed deionized water (pH 7.4) to prepare solutions of 1.25% (*w*/*v*) and 6% (*w*/*v*), respectively. Final component concentrations within 150 µL hydrogels were 0.8% *w*/*v* for CMHA-S and 1.2% *w*/*v* for PEG-DA as detailed in Table 1. Hydrogels that incorporated HAse (50 U–250 U) were formulated in the same manner except that the HAse component was pre-mixed with the PEG-DA solution prior to adding the other components.

### 4.4. Tumor Model and Treatment with Anti-CTLA-4

The immunotherapeutic activity of anti-CTLA-4 in tumor-bearing mice was assessed as prescribed previously [13]. In brief, C57BL/6 mice were implanted with MC-38 cells (3 × 10^5^/mouse) by SQ injection into the right upper flank. For optical fluorescent imaging experiments, mice (2 mice/group) were treated with a single dose of anti-CTLA-4/Dylight 800 delivered by either IP injection (100 µg in PBS) or by peri-tumor SQ injection (25 or 50 µg) using a hydrogel formulation between 11 and 14 days after tumor implantation. For assessing the impact of hydrogel-encapsulated anti-CTLA-4 (50 µg/dose) on tumor growth, all animals were implanted with tumor on the same day and those with palpable tumors at Day 6 were randomly divided into treatment groups (N = 9–10/group). All three groups received a 150 µL injection of hydrogel-encapsulate anti-CTLA-4 delivered by peri-tumor SQ dosing on Days 6 and 11. One group received a standard formulation without HAse, one containing 50 U HAse, and one with 2500 U HAse. Tumor volumes were measured by calipers every 3 to 4 days up to day 28.

### 4.5. Optical Fluorescence Imaging

In vivo and ex vivo optical imaging were performed using an IVIS Lumina II system (Caliper Life Sciences, Inc.; Hopkinton, MA, USA) at the Crump Preclinical Imaging Technology Center at UCLA. In vivo whole-body imaging of mice was carried out under isoflurane anesthesia with serial assessments in the same animals. For ex vivo imaging of LN, LN were recovered by surgical resection in replicate cohorts of animals that had been euthanized at various time points (0–72 h) after administration of anti-CTLA-4/DyLight 800.

### 4.6. Statistical Evaluations

Biodistribution studies were performed in replicates, with all animals imaged under identical settings at the same session to facilitate comparison of fluorescent intensity, as indicated by a continuous red to yellow intensity spectrum. Where indicated, visual findings were quantitated by assessing the fluorescent emission (photons/second; 800 nm wavelength) within the region of interest. Mean values are represented. Tumor immunotherapy responses are presented by individual spaghetti plots showing measured tumor volume over time for each animal in each treatment group. Comparison between groups was carried out by a tumor-free survival analysis employing a log-rank test. Impact on tumor growth over time was compared between groups using an ANOVA (two-factors with replication). *p* < 0.05 was considered as significant.

## Figures and Tables

**Table 1:** Hydrogel composition.

Hydrogel Component	Final Concentration in a 150 µL Injection
CMHA-S	0.8% *w*/*v*
PEG-DA	1.2% *w*/*v*
Hyaluronidase (HAse)	0, 50, or 250 Units

## Data Availability

The data presented in this study are available on request from the corresponding author.

## Supplementary Materials

The following supporting information can be downloaded at: https://www.mdpi.com/article/10.3390/gels8050284/s1, Figure S1: Effect of hyaluronidase (HAse) on the release of fluorescent-labeled anti-CTLA-4 from hydrogels; Figure S2: Inclusion of HAse within the hydrogel formulation enhances tumor-free survival; Table S1: Impact of hyaluronidase (HAse) on the delivery of hydrogel-encapsulated anti-CTLA-4 to TDLN.
