# Supplementary material for: Hyaluronidase Enhances Targeting of Hydrogel-Encapsulated Anti-CTLA-4 to Tumor Draining Lymph Nodes and Improves Anti-Tumor Efficacy
Source: Gels. 2022 May 3;8(5):284. doi: 10.3390/gels8050284 (PMC9141760; doi:10.3390/gels8050284)
Supplement: Supplementary file 1 [file gels-08-00284-s001.zip › gels-1658919-supplementary.pdf]

gels MDPI

Supplementary Materials

Hyaluronidase Enhances Targeting of Hydrogel-Encapsulated Anti-CTLA-4 to Tumor Draining Lymph Nodes and Improves Anti-Tumor Efficacy

Airi Harui * and Michael D. Roth

Table S1. Impact of hyaluronidase (HAse) on the delivery of hydrogel-encapsulated anti-CTLA-4 to TDLN. C57BL/6 mice bearing palpable MC-38 tumors were treated with 50 µg of hydrogel-encapsulated anti-CTLA-4/DyLight 800 by peri-tumor SQ injection. Anti-CTLA-4 biodistribution was compared in animals receiving a standard hydrogel formulation to one that incorporated HAse (250 U). Axillary TDLN were surgically-resected at 24, 48, 72 hrs from a cohort of animals treated in the same manner and subjected to ex vivo optical fluorescence imaging. Total emitted photons/second (×10^8) from anti-CTLA-4/DyLight 800 are presented.

Table 108.	Mean Fluorescence Emission (photons/sec) from recovered Axillary TDLN [×10^8]		
	24 h	48 h	72 h
Hydrogel	3.19	6.95	0
Hydrogel + HAse	12.8	6.89	1.18

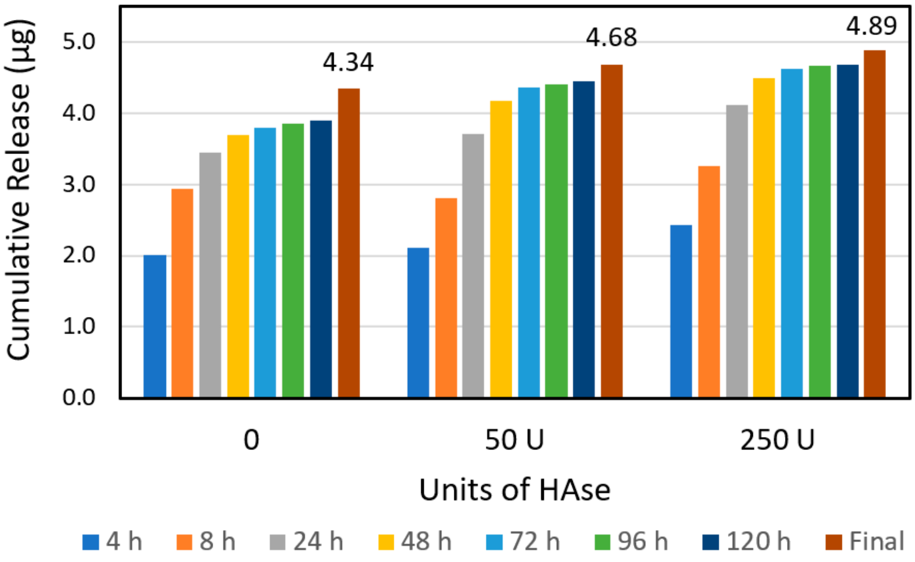

Figure S1. Effect of hyaluronidase (HAse) on the release of fluorescent-labeled anti-CTLA-4 from hydrogels. Hyaluronic acid hydrogels containing anti-CTLA-4/FITC (5µg) and HAse (0, 50, 250 units) were prepared. Hydrogels were incubated in release media at 37 °C with the media collected for analysis and replaced with fresh media at indicated time points. HAse was added after 120 h to lyse any residual hydrogel and release any retained antibody for "Final" measurement. Recovery of anti-CTLA-4/FITC at each time point was measured using a

fluorescence plate reader. A HAse dose-dependent effect was observed on the rate of release and on the overall recovery efficiency.

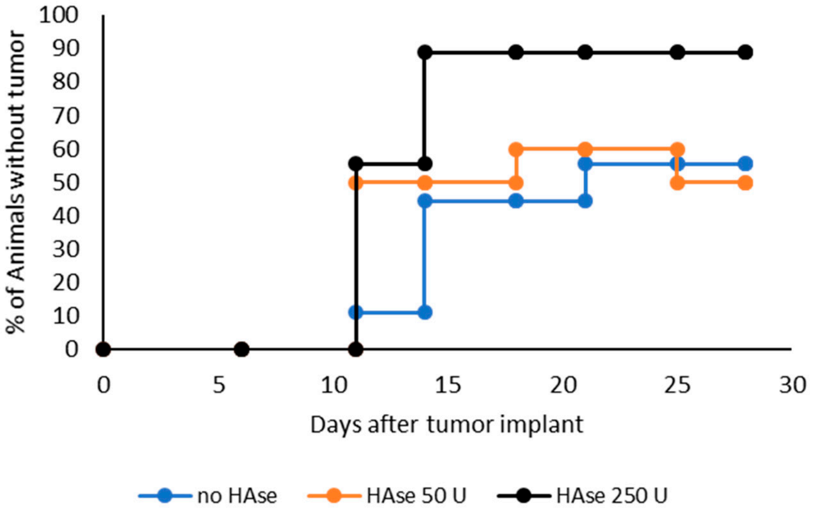

Figure S2. Inclusion of HAse within the hydrogel formulation enhances tumor-free survival. C57BL/6 mice with palpable subcutaneous MC-38 tumors were treated with hydrogel-encapsulated anti-CTLA-4 (50 μg) and HAse (0, 50, or 250 units) by peri-tumor SQ injection at days 6 and 11 after tumor implantation. % of tumor free mice at each time point are presented. *$p<0.05$ compared to no HAse control by log-rank test.
